# Past, present, and future developments in enantioselective analysis using capillary electromigration techniques

**DOI:** 10.1002/elps.202000151

**Published:** 2020-09-28

**Authors:** Nicky de Koster, Charles P. Clark, Isabelle Kohler

**Affiliations:** ^1^ Leiden Academic Centre for Drug Research, Division of Systems Biomedicine and Pharmacology Leiden University Leiden The Netherlands; ^2^ Division of BioAnalytical Chemistry, Department of Chemistry and Pharmaceutical Sciences, Amsterdam Institute for Molecular and Life Sciences Vrije Universiteit Amsterdam Amsterdam The Netherlands

**Keywords:** CE‐MS, Chiral analysis, Chiral CEC, Chiral electrokinetic chromatography, Enantioselective separation

## Abstract

Enantioseparation of chiral products has become increasingly important in a large diversity of academic and industrial applications. The separation of chiral compounds is inherently challenging and thus requires a suitable analytical technique that can achieve high resolution and sensitivity. In this context, CE has shown remarkable results so far. Chiral CE offers an orthogonal enantioselectivity and is typically considered less costly than chromatographic techniques, since only minute amounts of chiral selectors are needed. Several CE approaches have been developed for chiral analysis, including chiral EKC and chiral CEC. Enantioseparations by EKC benefit from the wide variety of possible pseudostationary phases that can be employed. Chiral CEC, on the other hand, combines chromatographic separation principles with the bulk fluid movement of CE, benefitting from reduced band broadening as compared to pressure‐driven systems. Although UV detection is conventionally used for these approaches, MS can also be considered. CE‐MS represents a promising alternative due to the increased sensitivity and selectivity, enabling the chiral analysis of complex samples. The potential contamination of the MS ion source in EKC‐MS can be overcome using partial‐filling and counter‐migration techniques. However, chiral analysis using monolithic and open‐tubular CEC‐MS awaits additional method validation and a dedicated commercial interface. Further efforts in chiral CE are expected toward the improvement of existing techniques, the development of novel pseudostationary phases, and establishing the use of chiral ionic liquids, molecular imprinted polymers, and metal‐organic frameworks. These developments will certainly foster the adoption of CE(‐MS) as a well‐established technique in routine chiral analysis.

**Abbreviations**
CMT: counter‐migration technique
IL: ionic liquid
MIP: molecularly imprinted polymer
MOF: metal‐organic framework
OT‐CEC: open‐tubular CEC
PFT: partial‐filling technique
PSP: pseudostationary phase

## 1 Introduction

It is not long ago that molecular chirality has been recognized to have profound implications for all living systems, despite having first been described by Pasteur in 1848 [1, 2]. Chiral molecules exist in two mirror‐image isomers called enantiomers. While being identical in their connectivity, enantiomers differ in their overall shape due to a different spatial orientation of atoms within the molecule. Even with this apparent minor difference, chirality has a significant impact on nature [3, 4, 5, 6, 7, 8, 9], as illustrated by the chiral growth of biological structures [3, 4], the existence of many chiral signaling molecules such as pheromones [5, 6, 7], flavors [8] and odors [9], as well as the stereospecificity of many physiologicallyrelevant biochemical reactions.

Due to the difference in biological activities frequently observed between two enantiomers, controlling chirality has become increasingly important in many pharmaceutical and industrial applications. Indeed, the separation and characterization of enantiomers are now considered an essential step in the drug development pipeline. The thalidomide tragedy in the 1960s shed the light on the importance of chirality in drug efficacy and safety, as both enantiomers and their respective metabolites can have substantial differences in their pharmacological and/or toxicological properties [10, 11]. The (*R*)‐enantiomer of thalidomide has sedative properties while the (*S*)‐isomer is teratogenic. Administration of the thalidomide racemate to pregnant women to treat morning sickness led to numbers of birth defects (typically phocomelia) and stillborn babies. Nowadays, pharmacological and toxicological data are required during the drug development process to grant market access to newly developed drugs, a process strictly controlled by government regulations. Besides pharmaceutical industries, the characterization of enantiomers has also become relevant in other fields of research, such as forensic science, metabolomics, food analysis, the agrochemical industry, and environmental analysis [12, 13, 14, 15, 16, 17].

Enantiomers of chiral molecules possess identical physicochemical properties and molecular mass, and typically cannot be separated using conventional analytical techniques. Highly enantioselective separation approaches are therefore needed, which are conventionally either based on the use of chiral columns, the derivatization of both enantiomers, or the use of a chiral selector. A large diversity of analytical strategies have been reported so far, mostly based on gas chromatography [18, 19, 20], liquid chromatography [21, 22, 23, 24, 25, 26, 27], and supercritical fluid chromatography [28, 29, 30, 31]. Next to these chromatographic approaches, CE represents an excellent alternative for chiral analysis. In chiral CE, a chiral selector is added to the BGE, forming a fast and reversible equilibrium with the enantiomers. The apparent mobilities of the enantiomers therefore depend on the equilibrium constant of the complex formed with the chiral selector, which differs between the two isomeric forms. This concept of chiral separations by CE was pioneered by Gassman et al. [32] in 1985, who separated enantiomers of derivatized amino acids via addition of a Cu(II) l‐histidine chiral support electrolyte.

Besides the typical beneficial features of CE (i.e., low solvent and sample consumption, simplicity, and orthogonality of the separation mechanism compared with chromatographic techniques), chiral CE also provides highly efficient separations at a reasonable cost, as only a limited amount of chiral selector is required. Moreover, many different chiral selectors can be tested rapidly, and the overall cost is significantly less than purchasing a dedicated chiral chromatographic column. The enantiomeric separation is also usually faster in CE compared with chromatographic techniques. For all of these reasons, chiral CE is therefore considered an attractive tool for the separation of enantiomers. Consequently, extensive research in recent years has resulted in the establishment of several chiral CE approaches, including chiral EKC and chiral CEC. EKC and CEC both rely on the EOF for pumping the mobile phase through the capillary or column, benefitting from the flat flow profile and reduced band broadening. CEC, however, uses either packed columns, monoliths, or coated capillaries to immobilize the chiral selector molecules, as opposed to adding them to the BGE.

In applications of chiral CE where sensitivity is not crucial, ultraviolet/DAD (CE‐UV/DAD) is the preferred method. However, in many cases—mostly when analyzing complex biofluids or very diluted samples—the sensitivity observed with UV is not sufficient. In this context, CE can be hyphenated to MS, allowing for a significantly increased selectivity and sensitivity, and providing additional information on compound identity. Over the last decade, CE‐MS has been demonstrated as an attractive technique for a large diversity of applications, including the analysis of pharmaceuticals [33, 34, 35], endogenous metabolites and biomarker candidates [36, 37, 38, 39, 40], (intact) proteins and peptides [41, 42, 43, 44], carbohydrates [45, 46, 47], food and environmental pollutants [48, 49, 50, 51], and single cells [52, 53]. Due to its relatively higher practical complexity, CE‐MS is not used as often as other MS‐based chromatographic techniques. Nevertheless, CE‐MS has started to become a more established technique. Consequently, extensive research on the development of MS‐compatible CE conditions has also fostered the development of chiral CE‐MS [54]. In this context, efforts have mainly focused on chiral EKC‐MS and chiral CEC‐MS approaches, with the development of suitable interfaces, MS‐compatible experimental conditions, novel chiral selectors, and chiral stationary phases.

This review discusses the state‐of‐the‐art approaches and recent developments carried out in the field of chiral CE, focusing on chiral EKC and chiral CEC. In addition to the well‐established chiral selectors and CEC stationary phases, novel approaches involving the use of chiral ionic liquids (ILs), molecularly imprinted polymers (MIPs), and metal‐organic frameworks (MOFs) are described. The final section of this review is dedicated to the coupling of CE to MS in the context of chiral applications.

## 2 Chiral EKC

In chiral EKC, enantiomers are separated based on the dynamic equilibrium formed with a pseudostationary phase (PSP) added to the BGE. The formation of distinct noncovalent enantiomer–PSP complexes effectively limits their mobility to the point where the separation between the two enantiomers is achieved. Many different structures can adopt the role of PSP, for example, CDs, crown ethers, and antibiotics, provided that their functionality and stability is retained in the BGE and that they are compatible with the detection method. Both unimolecular chiral selectors and supramolecular structures can function as PSPs, the latter being used in MEKC. Novel chiral selectors are still being developed; among them, chiral ILs show great promise through their versatility and observed synergistic effects with other chiral selectors. Additionally, the interaction between the chiral analyte and various PSPs can be further enhanced by chemical derivatization.

### 2.1 Chiral selectors

#### 2.1.1 Cyclodextrins

CDs belong to the earliest selectors implemented for chiral CE separations and remain the most frequently used enantioselector in chiral EKC. Their implementation as a chiral selector was first demonstrated by Snopek et al. in 1988 [55], who used β‐CDs as the leading electrolyte during isotachophoresis. That pioneering work was built upon by Fanali et al. [56] who showed in 1989 that β‐CDs can be used in chiral CE separations, applied to the separation of sympathomimetic drugs.

**Figure 1:**
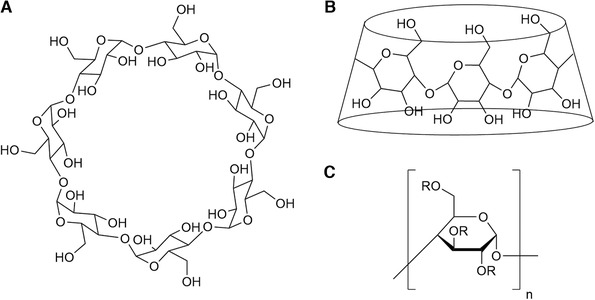
Molecular structure of a β‐CD. (A) The seven glucose units connect through ether bonds to form the β‐CD. (B) Three‐dimensional view of a CD. The cyclic structure adopts a truncated cone shape with primary and secondary hydroxyl groups presented outward. Analytes approach the CD core from beneath, where chiral recognition occurs at the cavity mouth. (C) Each hydroxyl group can be substituted for a desired functionality (R).

CDs are cyclic oligosaccharides consisting of glucopyranose monomers joined by α‐1,4‐glycosidic bonds (Fig. 1A). Within their hydrophobic cavity, a wide range of chiral organic molecules can form noncovalent interactions within the extensive chiral environment of the glucopyranose functionalities, giving distinct diastereoisomeric complexes that differ in their host–guest orientation. In an aqueous medium, the chiral analyte replaces the water molecules present in the CD core via an inclusion mechanism [57], driven by the difference in polarity between the BGE, the analyte, and the selector, and possibly by additional interactions between the analyte and the functional groups along the CD exterior. The consequent enantioseparation depends on both the difference in thermodynamic stability of the respective host–guest complexes as well as the difference in their effective mobility [58, 59]. A detailed mathematical description of the CD–analyte complexation can be found in a specialized review on the topic [60]. However, some have argued against the assumption of inclusion as a requisite for chiral recognition [61, 62], since in selected instances enantioselectivity was achieved through other mechanisms (e.g., external complexes) [63, 64], leaving the complexation mechanism of the full spectrum of CD derivatives to be only partly understood. Nonetheless, molecular modeling and NMR experiments, in combination with EKC separations, have significantly contributed to the understanding of the mechanisms underlying the chiral recognition of CDs, and continuous efforts are expected to further extend this knowledge in the future [60, 65, 66].

CD enantioselectivity can be further tailored by optimizing the size and substitution patterns of the selector. Primarily, CDs composed of six, seven, or eight glucose units are employed, referred to as α‐, β‐, and γ‐CDs, respectively. The variety in cavity dimensions and possible functional groups on the edges of the adopted truncated cone shape of CDs (Fig. 1B and 1C) allows for the recognition of a wide range of chiral compounds [65]. Functionalities on the CD rim can often contribute to the chiral recognition through supplying complementary binding sites for increased complex stability [67]. Furthermore, they can provide CDs with an adequate electrophoretic mobility [68] and increase their solubility in aqueous media; native CDs, especially native β‐CDs, only have a limited solubility [69]. Overall, the large diversity of possible derivatives leads to a high potential for selectivity and resolution.

Larger cycles have not been studied as extensively, mainly because they are more difficult to isolate and purify from enzymatically degraded starch than their smaller analogues [70], and due to their reputation of having negligible complex forming capacity due to their increased size and flexibility [65]. However, several studies published over the last 20 years have supported the potential of larger CDs for enantioseparations [70, 71, 72, 73, 74, 75]. For instance, molecular dynamics studies of ranges of large‐ring CDs suggest they can adopt a dominant preferred conformation [72, 73], that is, they do not inherently possess a decreased chiral recognition ability due to continuous conformational changes in solution allowed by their supposed highly flexible structure. Furthermore, micromolar affinities toward larger CDs have been observed for engineered hosts [74]. Recent advances in manipulating the product specificity of CD glucanotransferase toward large rings should foster further developments in the use of these unconventional hosts [76, 77].

The use of CDs for enantioselective separation has become a well‐established approach in electromigration, as highlighted by the number of new CD derivatives reported over the last two decades [61]. The compatibility of CDs with aqueous buffers, their stability in solution, and low toxicity [78] also represent interesting practical and environmental advantages. The use of CDs has also been demonstrated for the separation of very low amounts of enantiomeric impurities. For instance, by employing (2‐hydroxypropyl)‐β‐CD, Sánchez‐López et al. achieved the analysis of 0.02% of *R*‐duloxetine, separated from *S*‐duloxetine, with a LOD of 20 ng/mL [79]. Furthermore, CDs can separate chiral analytes and their respective chiral metabolites within the same run [80]. One potential drawback of CDs is a relatively lower selectivity compared to more rigid chiral selectors due to a possible induced‐fit mechanism [61]. However, such drawbacks, regardless of the chiral selector employed, are usually compensated by the high efficiencies observed in CE.

With the established advantages of employing CDs in CE and the commercial availability of derivatives, novel CD systems have been continuously developed and applied to diverse fields. Currently, both single and dual selector systems are employed. Single CDs can differ in their degree of substitution, namely, they are either randomly derivatized or present as a single isomer. Randomly derivatized CDs have an average number of substitutions; in spite of having their own selectivity profile, the reproducibility is relatively poor [65]. For single‐isomer CDs, typically all hydroxyl groups are substituted with the same function [65], while monosubstitution is less common due to a more complex synthesis. Although a trend toward using single‐isomer CDs has been observed [61, 65], it is nonetheless recommended to start with statistically substituted CDs before employing single‐isomer variants as they are not necessarily required for a successful enantioseparation.

#### 2.1.2 Crown ethers

Crown ethers are interconnected polyethers that can recognize cations by host–guest complexation of the analyte with their oxygen atoms via ion‐dipole interactions and/or hydrogen bonds [81, 82]. These compounds were implemented as a chiral stationary phase in liquid chromatography separations as early as 1987 [83], before Kuhn et al. used an optically active crown ether as the chiral selector for CE separation of amino acids in 1992 [84]. Chiral separation is achieved by substituting the ethylene bridges, which can also provide an additional stabilization of the complex. Due to this mechanism of binding, the application of crown ethers is limited to enantiomers carrying primary amines, including aromatic amines, amino alcohols, and amino acids. The strongly acidic conditions required to obtain fully protonated amines and the exclusion of competing BGE cationic species further limit their scope.

The work by Kuhn et al. demonstrated that chiral resolution is based on the stability of diastereoisomeric complexes between crown ethers and analytes, highlighting the importance of optimizing the crown ether concentration, buffer pH and composition, and temperature for separations [85, 86]. Subsequently, Walbroehl et al. compared the use of a chiral crown in the BGE in chiral CE or the incorporation of a chiral ether in a stationary phase in HPLC. The two techniques were found to be complementary for the separation of amines [87, 88]. Crown ethers have since been employed as the sole chiral selector in enantioselective CE, finding application in, for example, the analysis of pharmaceuticals [89, 90, 91], psychoactive substances [92, 93], and amino acids [94]. Among the different available chiral crown ethers, (+)‐(18‐crown‐6)‐2,3,11,12‐tetracarboxylic acid remains the most commonly used variant.

**Figure 2:**
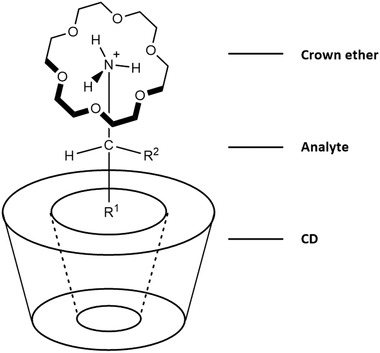
Supramolecular interaction between a crown ether, cationic analyte, and a CD. Suggestion of the molecular model (schematic) of the complexation of a 18‐crown‐6, a primary amino compound, and a β‐CD. Chiral recognition can be enhanced by using this dual selector system. Adapted from [96] with permissions.

In many cases, however, employing crown ethers as the sole chiral selector is insufficient for complete enantioseparation, and they may show more potential in dual selector systems, enhancing the chiral recognition as well as the resolution and sensitivity [95]. For example, when combined with CDs, crown ethers can enhance chiral recognition by sandwiching the analyte between them and the CD core (Fig. 2) [82, 96, 97, 98]. In some cases the addition of a crown ether is essential, as no enantioseparation is observed using CDs only [99, 100]. The synergistic effects of employing CDs and crown ethers simultaneously were already acknowledged in early studies [101], and their combination has since been a viable strategy in chiral separations [102, 103, 104, 105, 106]. If chiral recognition would originate fully from the applied CD, less expensive achiral crown ethers can also be added for improved stability.

#### 2.1.3 Antibiotics

The performance obtained with CDs and crown ethers has fostered the investigation of other naturally occurring macrocycles, resulting in the implementation of antibiotics as novel chiral selectors. The interest in these structures is reflected by the numerous reviews written on this topic [107, 108, 109, 110, 111, 112, 113, 114, 115]. Antibiotics possess many chiral centers and different functionalities which are beneficial for molecular interactions with enantiomers, and are suited for the chiral separation of a broad range of compounds. Since their solubility is structure dependent, antibiotics are applicable as chiral selectors to both aqueous as well as nonaqueous approaches. The first usage of macrocyclic antibiotics as chiral selector molecules was introduced by Armstrong et al. in 1994 [116], prior to which the majority of chiral CE separations relied on CDs. Five classes of antibiotics are now commonly used for enantioseparation, including macrocyclic antibiotics, more simple interconnected structures, and noncyclic selectors, as listed in Table 1 [117, 118, 119, 120, 121, 122, 123, 124, 125, 126, 127, 128, 129, 130, 131, 132, 133, 134]. Some antibiotic classes are more suited for the separation of compounds that possess specific physicochemical properties, for example, glycopeptides are often used for the separation of acidic analytes, while basic compounds are best recognized by ansamycins [135]. The exact chiral recognition mechanism has, however, not been fully elucidated [114].

**Table 1:** Different types of antibiotics used in chiral analysis

Antibiotic class	Structural characteristics^a)^	Antibiotic	Analytes	Analyte properties^b)^	Reference
Glycopeptides	Multiple macrocycles, each cycle containing two aromatic rings	Avoparcin	N‐blocked AAs	Acidic	[117]
		Balhimycin	N‐blocked AAs		[118]
		Bromobalhimycin	N‐blocked AAs		[119]
		Eremomycin	N‐blocked AAs, profens		[120, 121]
		Ristocetin	120 acidic compounds		[122]
		Teicoplanin	N‐blocked AAs		[123]
		Vancomycine	FMOC‐derivatized AAs		[124]
Ansamycines	Single macrocycle with naphthahydro‐quinone ring	Rifampicin	Mixed	Mixed, primarily basic	[125]
		Rifamycin	Basic drugs		[126]
Macrolides	Single macrocyclic lactone with sugar monomer side chains	Azithromycin	Basic drugs, tryptophan	Basic, zwitterionic	[127]
		Boromycin	Primary amines (basic)		[128]
		Clarithromycin	Basic drugs		[129]
		Erythromycin	Basic drugs		[130, 131]
Lincosamides	No macrocycle, consecutively contains a amine, an amide, and a sugar moiety	Clindamycin	Basic drugs	Basic	[132]
Aminoglycosides	No macrocycle, multiple interconnected glycosidic rings, functionalized with nitrogen atoms	Fradiomycin	Acidic drugs	Acidic and basic	[133]
		Kanamycin	Acidic drugs		[133]
		Streptomycin	Acidic drugs		[134]
Miscellaneous	‐	β‐Lactams	Basic drugs	Basic	[133]

^a)^ Obtained from [114].

^b)^ Analytes categorized according to the relevance of their acidity and basicity for their analysis. AA, amino acid; FMOC, fluorenylmethyloxycarbonyl.

Various experimental conditions can affect chiral separation using antibiotics. First, the concentration of the antibiotic chiral selector must be adjusted and the composition of the BGE has to be carefully optimized. Organic modifiers such as methanol or ethylene glycol are often required to ensure the solubility of the antibiotic, which can significantly alter the enantioresolution [114]. Furthermore, the capillary should be maintained at a temperature between 15 and 25°C to avoid antibiotic precipitation [114]. Several constraints have been associated with the use of antibiotics in CE, including self‐aggregation and adsorption to the capillary wall [78, 136, 137]. The latter can be reduced by optimizing the washing procedure or by modifying the BGE, or it can be prevented by using coated capillaries, as discussed by Dixit et al. [114].

One early example of chiral CE with antibiotic chiral selectors comes from Vespalec et al., who used submillimolar concentrations of vancomycin added to the BGE to achieve separation of amino acid enantiomers in less than 5 min [138]. In a more recent work, a dynamic coating of poly(dimethylacrylamide) has been added to the capillary, minimizing the adsorption of vancomycin to the capillary wall [139]. Other groups have continued to push research into novel chiral selectors from different classes of antibiotics, including boromycin [] and rifampicin [125]. Additionally, there have also been recent reports of synergistic effects of the antibiotics clarithromycin lactobionate [129] and clindamycin phosphate [132] with other chiral selectors in dual selectors systems.

#### 2.1.4 Surfactants

In chiral MEKC, chiral recognition is based on the partitioning of the chiral analyte between a micellar PSP and the surrounding BGE [140]. Micelles are formed upon adding surfactant monomers to an aqueous solution above their CMC. Enantiomers can partially solubilize within these dynamic aggregates by complexation; the mechanism of their enantioseparation is similar to that of chiral selectors. The first documented study that used this principle for chiral electrokinetic separations was performed by Terabe et al. in 1989 [141], who showed that bile salts (a chiral surfactant) could be used to separate racemic amino acid mixtures.

Extensive research in the following decades led to a better understanding of the use of surfactants in chiral MEKC, as well as tools to understand and predict the interactions between enantiomers and the micellar phase. Monomeric surfactants should be sufficiently soluble in solution to form micelles above their required CMC. For instance, some conventional chiral surfactants like sodium cholate and sodium deoxycholate have CMCs of 14 mM and 7 mM, respectively [142, 143]. Moreover, they should form a homogeneous micellar phase throughout the BGE that is compatible with the method of detection and have a low viscosity [144]. The enantioselectivity is determined by the type of interactions that take place, which are based on the properties of the analyte [140]. The solute either interacts with the surface of the micelle via electrostatic or dipole interactions, acts as a co‐surfactant and incorporates itself into the supramolecular micelle structure, or partitions into the hydrophobic interior of the micelle. To achieve enantioseparation in MEKC, systems using a single type of chiral surfactant or mixtures of both chiral and achiral surfactants can be employed. Chiral selectors are also often combined with micellar PSPs to enhance separation power, which is considered one of the most effective approaches for achieving enantiomeric separations by MEKC [140]. Following this strategy, both chiral as well as achiral micelles can be employed.

Nowadays, the most frequently used surfactants suitable for chiral MEKC are chiral bile salts, polymeric (molecular) micelles, and achiral surfactants such as SDS that are used in combination with a chiral selector [140]. MEKC using chiral bile salts benefits from their commercial availability [145]; mainly sodium cholate, sodium deoxycholate, sodium taurocholate, and sodium deoxytaurocholate have been employed [140]. The use of other nonbile chiral surfactants has also been successfully reported, including amino acid based surfactants [146] and vesicle‐forming cationic surfactants [147]. Additionally, a trend has been observed in recent years toward the implementation of polymeric micelles in chiral MEKC [140]. Furthermore, mixtures of chiral and achiral micellar systems have proven to be very useful, including those based on mixed micelles of SDS and bile salts [148], as well as mixed polymeric micelles [149]. These combined systems may offer many possibilities for improved enantioseparations of a broad range of chiral compounds.

The combination of a chiral selector with micelle‐based separations has also become increasingly common, with many examples of work combining β‐CDs with bile salts or SDS [150, 151, 152, 153]. The addition of CDs to a chiral MEKC separation results in a two PSPs system, which can further increase resolution [154]. As an example, Ibrahim et al. found that a combination of either of two CDs with MEKC based on SDS provided better separation of imidazole enantiomers than any PSP individually [153]. Furthermore, many studies have reported ligand‐exchange MEKC, another variant to achieve chiral separation [155, 156, 157]. This technique is intriguing for its ability to enhance separation resolution as well as influence enantiomer migration order [158]. Innovative work from Zaher et al. showed that a lipophilic species could serve as both the central ion‐complexing ligand and the micelle‐forming surfactant, which they demonstrated with amino acid separations [159].

Taking advantage of these extensive developments, chiral MEKC has since been applied in the analysis of food, biological samples, pharmaceuticals, and chiral analytes relevant in forensic and environmental analysis, among other categories [140]. For a comprehensive summary of the current status of MEKC and its future potential, the reader is referred to a recent review from 2020 by Salido‐Fortuna et al., where MEKC enantioseparation principles, separation strategies, mechanistic studies on chiral recognition, preconcentration techniques, and its application in the analysis of real samples are discussed [140].

#### 2.1.5 Chiral ionic liquids

ILs are salts of bulky nonsymmetrical organic cations that are typically liquid at ambient temperature. The wide versatility in their structure and physicochemical properties make them highly attractive in analytical chemistry, which is reflected in the many reviews written on their application in separation science [160, 161, 162, 163, 164, 165]. IL components exert intermediate interionic forces, providing them with interesting properties that lie between those of organic solvents and inorganic salts, that is, high conductivity, liquid state at a wide temperature range, and thermal stability [160, 162, 166]. Furthermore, they have tunable viscosity, are highly miscible with aqueous as well as organic solvents, and have a low volatility [160, 161, 162, 166]. High separation efficiency and selectivity using ILs have been reported in both CE and HPLC applications [160].

**Figure 3:**
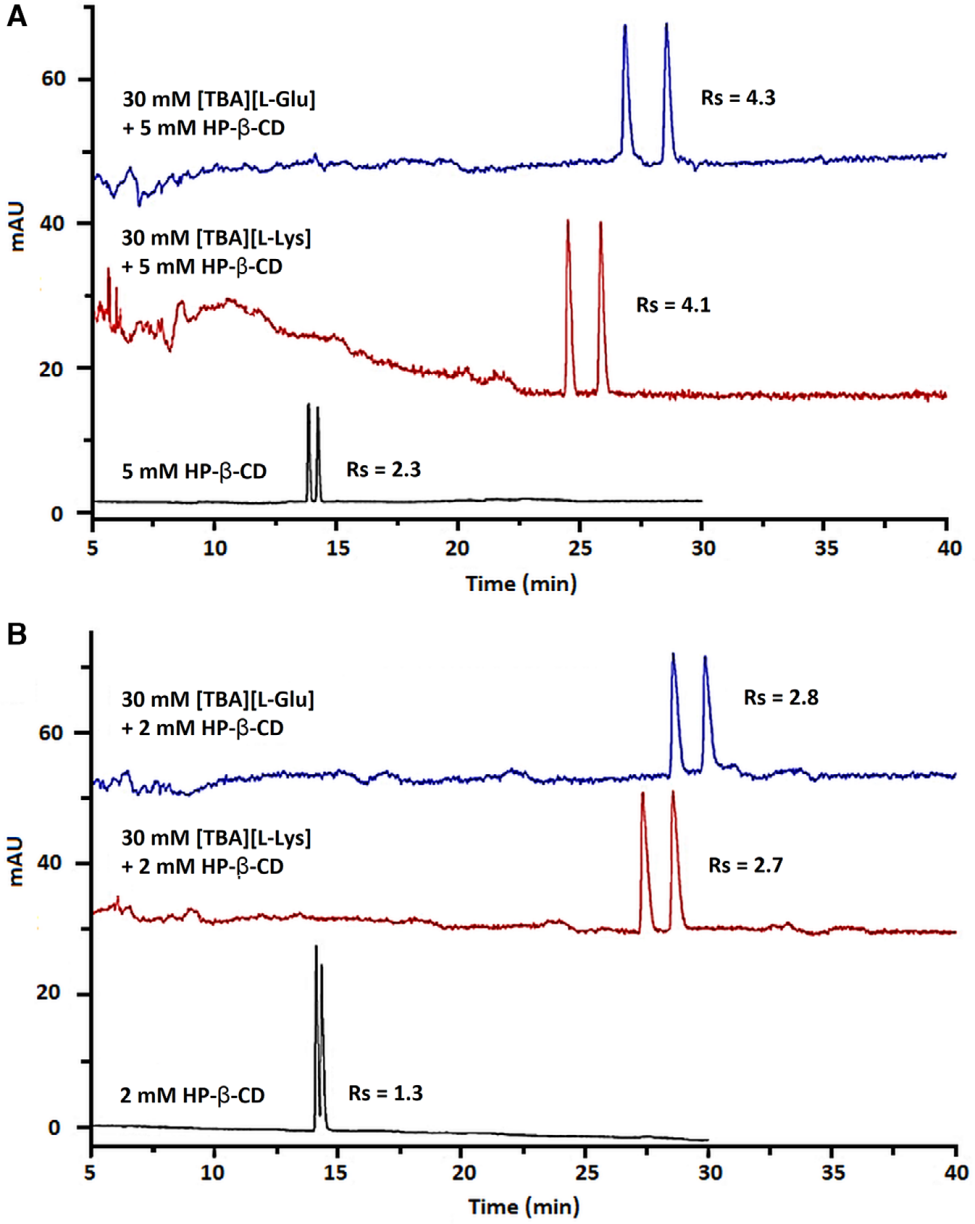
Synergistic effects of β‐CD and chiral ionic liquids in a dual selector system, illustrated with the enantioseparation of chiral drugs econazole (A) and sulconazole (B). The use of a single chiral selector, (2‐hydroxypropyl)‐β‐CD shown in the bottom trace, was compared to two dual selector systems including (2‐hydroxypropyl)‐β‐CD and either the chiral ionic liquid [TBA][L‐Glu] shown in the top trace, or [TBA][L‐Lys] shown in the middle trace. Enhanced resolution was observed for both chiral drugs when using a dual selector system, compared to only employing (2‐hydroxypropyl)‐β‐CD. Rs = resolution. Adapted from [170] with permissions.

In chiral analysis, ILs can have many functions, namely as a chiral selector, ligand, and stationary phase. Additionally, good electrical conductivity is obtained with ILs as supporting electrolyte or EOF modifier. In optimized conditions, ILs can fulfill multiple tasks simultaneously. Interactions of ILs with chiral analytes leads to baseline enantioseparation; often synergistic effects are observed in dual selector systems with additional PSPs, as illustrated in **Fig**. **3** [69, 167, 168, 169, 170]. As some ILs resemble surfactants, they can also be employed as PSP in chiral MEKC analysis [160, 161, 171]. Novel developments include the fabrication of monolithic IL columns and IL silica hybrids, as well as polymeric ILs that resemble molecular micelles [161].

Unfortunately, chiral ILs are not widely commercially available yet, limiting further developments. Also, synthetic routes and purification methods are still in their early development and require further optimization for improved material properties [161, 163]. Although chiral ILs are generally considered ecofriendly due to their nonvolatile nature, the impact of their improper disposal, environmental toxicity, and long‐term stability have not been fully investigated yet [160, 163, 164, 165]. Further research is therefore essential for a widespread use in chiral CE analysis.

### 2.2 Chiral separation by chemical derivatization

A different approach to chiral separation by EKC is to chemically convert enantiomers to their respective diastereoisomers by derivatization. Each enantiomer then displays a different electrophoretic mobility due to the modification of its physicochemical properties, allowing for greater separation between derivatized analytes. Furthermore, derivatization can enhance chiral recognition by increasing the hydrophobicity necessary for MEKC separations of more hydrophilic compounds [172], or improve the interactions with a chiral selector (e.g., CDs) [173]. The ability to introduce UV absorbing or fluorescent groups also represents a significant advantage [172, 174].

The range of chiral compounds that can be separated by this approach is mainly limited to their functional groups. The reaction times for each analyte‐derivatizing reagent combination can vary extensively (i.e., from several minutes to 24 h), while additional measures to remove the excess reagent and its by‐products can be necessary [175]. Many types of derivatizing reagents are available [172, 176], which are specific for a particular molecular functionality (e.g., amines, carbonyls, hydroxyls, thiol, and carboxylic acids). Also, various pre‐capillary, on‐line, and on‐capillary derivatization methods have been developed in the last 20 years [172, 176, 177, 178, 179, 180], facilitating further developments of this approach.

Enantiomers can react with both chiral as well as achiral derivatizing reagents to obtain diastereomers. The indirect approach using chiral derivatizing reagents has been praised for its simplicity [181], since enantioseparation can be achieved without the need for additional chiral selectors. However, chiral reagents with high enantiomeric purity are required, drastically increasing the costs. Therefore, achiral derivatizing reagents are also often employed.

The majority of chiral EKC studies reported which separated derivatized enantiomers have combined the derivatization with MEKC analysis, with the aid of an additional chiral selector (i.e., CD), using both chiral [174, 182, 183] as well as achiral [184, 185, 186, 187, 188, 189, 190, 191, 192] derivatizing reagents. In an early study, Mechref et al. derivatized herbicide compounds with fluorescent tags, followed by CE with CDs and added micellar phases [185]. Compared to native compounds, the derivatized molecules showed better chiral recognition and achieved higher resolution. In recent years, one of the most commonly used derivatizing reagents has been FMOC, a popular choice as it yields stable derivatives from rapid reaction with amino compounds. Chiral separations employing FMOC derivatization typically use β‐CDs in combination with a surfactant, and have been applied extensively to separate amino acid mixtures [190, 192]. Compatibility of derivatization with technological advances, for example, MEKC separations with plastic microchips [178] and integrated sample pretreatment with in‐capillary derivatization for high‐throughput screening [177], was also demonstrated. Recent advances include novel derivatizing agents [193] and separations of FMOC‐derivatized amino acids with chiral ILs [168].

## 3 Chiral CEC

As opposed to the addition of a chiral selector to the BGE in EKC, chiral CEC uses chiral stationary phases to achieve enantioseparation. This hybrid technique relies on both the interaction of the analyte with the stationary phase, as well as on the bulk fluid pumping of the EOF. Consequently, the high efficiencies achieved with EKC due to the plug‐like flow profile are combined with the orthogonal separation mechanism of chromatography. The concept of CEC was first described in 1974 by Pretorius et al. [194], who demonstrated that EOF can be used to drive liquid through a packed column of stationary phase. This technique was improved upon by pioneering work from Jorgenson and Lukacs in 1981 [195], who used RP CEC to separate polyaromatic hydrocarbons. A decade later, the first demonstration of chiral CEC was provided by Mayer and Schurig [196], who coated their capillaries with a permethyl‐β‐CD‐modified dimethylpolysiloxane (Chirasil‐dex) for the separation of enantiomers.

Generally, there are two approaches for incorporating chirality into a CEC stationary phase. A chiral selector can either be covalently attached to an achiral stationary phase or dynamically coated onto the inner surface of the capillary column. Since covering the column support material can lead to a decreased EOF and thus increased migration times [197], a chiral selector that can provide sufficient EOF is essential, or the stationary phase should be left partially exposed. Since factors influencing an electricity‐driven bulk flow through a stationary phase are not yet completely understood, this remains challenging. A variety of selectors can be incorporated for chiral recognition, including polysaccharides, macrocyclic antibiotics, CDs, crown ethers, ion‐ and ligand‐exchangers, Pirkle‐type selectors, and (glyco)proteins [198, 199, 200]. These selectors are usually supported by silica, polyacrylamide gels, or methacrylate‐based polymers. Optimal enantioresolution and minimal band broadening caused by mass transfer are dependent on experimental conditions, notably pore size, injection volume, temperature, and flow rate [136, 201]. It is worth mentioning that the mass transfer kinetics are dependent on the type of stationary phase used [136]. Moreover, shifting from aqueous to organic solvents can significantly affect the enantioseparation, with sometimes better performance under nonaqueous conditions [201]. Lastly, dual selector systems can also be achieved by introducing an additional chiral selector to the mobile phase [202]. Many possible stationary phases have been explored so far; they are typically divided into three categories: chiral packing materials, monoliths, and thin films for open‐tubular CEC (OT‐CEC).

### 3.1 Chiral packed‐column CEC

In chiral packed‐column CEC, microparticles with chiral selectors present on their surface are tightly packed together in the capillary. The chiral selector can either be bound to the stationary phase during the packing process or added after packing. The stationary phase can be extended to fill the entire volume of the capillary. CEC allows for the use of smaller particle sizes compared to HPLC due to the absence of any back pressure, which also leads to less longitudinal diffusion [201]. Since the inner wall does not significantly contribute to the formation of the layer of counter cations, the geometry and morphology of the packing material as well as the BGE composition largely determine the EOF [201]. Nowadays, packing materials are not used as often as the other CEC approaches due to the encountered obstacles related to frit fabrication, clogging, and fragility of the columns [203]. Frits have long been associated with bubble formation, a non‐uniform EOF, and the adsorption of analytes [204]. Burning a detection window in the capillary (e.g., for UV detection) contributes to the fragility of the column [205]. A major development in packed‐column CEC, however, was the use of tapered capillaries, which obviated the need for frits. Lord et al. [206] were the first to use externally tapered capillaries, while Choudhary et al. [207] first employed capillaries with an internal taper.

Applications of chiral stationary phases employing polysaccharides, macrocyclic antibiotics, CD derivatives, crown ethers, (glyco)proteins, Pirkle‐type selectors, ion‐ and ligand‐exchange selectors, and others can be found in specialized reviews on chiral CEC [136, 198, 204, 208, 209, 210]. Researchers who employ chiral packed‐column CEC often choose polysaccharide‐based chiral stationary phases due to their excellent efficiency, broad chiral selectivity, and overall stability [210]. The coating of packing materials with neutral polysaccharides does results in relatively long analysis times; however, many different techniques and novel related chiral stationary phases have been developed to counteract this disadvantage [211]. Some early examples of this approach include Otsuka et al., who used HPLC chiral stationary phase in a CEC separation to resolve racemic drug mixtures [212], as well as Girod et al., who employed a cellulose stationary phase with high chiral recognition, allowing for reduced concentration of the selector on the silica surface [213]. There have also been methods reported for enhancing the EOF through the packed column. Mayer et al. used a derivatized cellulose stationary phase to increase the EOF, thus decreasing the analysis time [214]. Chen et al. chose to add a positively charged spacer reagent to their immobilized chiral selector, generating high EOF for the separation [215]. More recently, Aturki et al. separated cathinone derivatives using a chiral stationary phase based on amylose tris(5‐chloro‐2‐methylphenylcarbamate), achieving LODs in the range of 25–100 ng/mL.

### 3.2 Chiral monolithic CEC

Chiral monolithic columns have been developed as an alternative stationary phase for CEC to overcome the challenges associated with packing materials. Monoliths exist as a single continuous porous structure fixed within the separation capillary, which eliminates the need for any frits or tapering. Moreover, the straightforward fabrication and high mechanical stability of monolith columns make them attractive in terms of practicality and routine use. New types of monoliths and the implementation of standard procedures for CEC analysis currently represent the major focus [216], using monolithic columns initially developed for HPLC analysis [204]. In an early study, three times higher column efficiencies were obtained by CEC compared to micro‐LC using the same CD‐modified monolithic column [217]. A recent review by Gama et al. discusses the contemporary synthetic routes, functionalization, and innovative applications of monoliths [218]. A review specifically covering the advances in enantiomeric resolution on monolithic chiral stationary phases is also available [219].

Monoliths require chiral features for enantioselective analysis. In general, these are generated by modifying a silica or organic polymer base either by one‐step copolymerization with a chiral monomer or by postpolymerization modification with a chiral selector. Generally, monolithic CEC benefits from the wide choice of available polymer monomers [220]. Additionally, monoliths can also be dynamically coated with a variety of chiral selectors, expanding their synthetic options. Chiral monolithic CEC has been performed using a wide array of chiral selectors. Kato et al. developed a protein‐encapsulation technique for preparation of monolithic columns with protein chiral selectors, a popular choice for monolithic CEC [221]. Polysaccharide stationary phases are also employed, for example in separation of warfarin enantiomers [222]. Macrocyclic antibiotics are also used as chiral selectors for monolithic CEC, and have the benefit of simplified monolith fabrication [223, 224]. Finally, CDs remain a popular chiral selector for monolithic CEC as well. As reported by Wistuba et al., chiral monolithic CEC with immobilized permethyl‐β‐CD‐silica demonstrated up to 100 000 theoretical plates, and showed a twofold higher column efficiency than LC employing the same column [225].

Although silica‐based monoliths exhibit the advantages of high mechanical strength, thermal stability and chemical durability [226], they can suffer from cracking during or after fabrication as well as poorly reproducible chiral functionalization [136, 227]. On the other hand, polymer‐based monoliths can be employed at a wider pH range, but are prone to swelling and shrinking in organic solvents [228]. In this context, organic‐silica hybrid monoliths may represent a compromise between silica‐ and polymer‐based columns, since an increased mechanical and morphological stability and satisfactory column performance have been observed [216, 229, 230]. Additionally, zirconia‐based monoliths have been proposed as possible alternatives. They are stable at any pH and can withstand high temperatures [204]. As an example, Zhou et al. recently prepared a sulfobutylether CD‐silica hybrid monolithic column by a sol‐gel “one‐step” synthetic method. Of the 26 racemic chiral compounds enantioseparated, 17 reached baseline separation after optimization of experimental conditions. The developed method has been suggested to be used in the future to prepare other CD‐functionalized monoliths. Furthermore, the preparation of a carbamoylated azithromycin‐incorporated zirconia hybrid monolithic capillary column was reported by Dixit et al., as a continuation of their series of hybrid monoliths containing antibiotic chiral selectors [231]. Long‐term stability and satisfactory reproducibility of the column fabrication procedure were observed.

### 3.3 Chiral open‐tubular CEC

In OT‐CEC, a sole thin layer of a stationary phase is either physically coated on or chemically bonded to the surface of the separation capillary. Instead of completely filling the capillary, the majority of the capillary is kept open for a free flow of the BGE, resulting in decreased analysis times. The more straightforward preparation of the stationary phase and operation of the system represent additional advantages of this CEC approach [199, 232]. Recently, a variety of methods have become available to prepare and functionalize OT‐CEC capillaries [233].

Liu et al. have applied chiral OT‐CEC using adsorbed avidin as the stationary phase for the analysis of ibuprofen, warfarin, and other drugs [234]. However, low separation efficiency was reported for some analytes, suggesting that this stationary phase should be used only for enantiomers known to have strong interactions with avidin. More recently, Aydogan et al. developed a novel open‐tubular zwitterionic column with polymer stationary phase, and showed ligand exchange chiral OT‐CEC [235]. Their work was validated by separation of six amino acid enantiomers. Finally, Zhang et al. reported in 2019 the development of chiral OT‐CEC with a stationary phase of modified β‐CDs conjugated to gold nanoparticles [236]. After optimizing the conditions, they showed the enantioselective separation of tramadol hydrochloride and zopiclone with high resolution.

The relatively low column capacity and limited resolution of chiral OT‐CEC remain disadvantageous for most of the applications. Longer capillaries are often needed, resulting in increased migration times, and there is a risk of overloading [204]. The current efforts focus on the development of novel capillary coatings to increase the total surface area and, thus, the recognition abilities of the stationary phase [237].

### 3.4 Novel chiral stationary phases for CEC

#### 3.4.1 Molecularly imprinted polymers

Molecular imprinting is an orthogonal fabrication method of chiral stationary phases employed in monolithic and OT‐CEC. The selectivity of MIPs is defined during the polymerization procedure by using a chiral template, leading to a cavity that specifically recognizes molecules with a similar shape to that of the template [204]. MIPs can usually only be used for separating the racemate of the template molecule, however with a very high resolution. Although many reviews on the fabrication of MIPs have been published that acknowledge their potential [238, 239, 240, 241, 242, 243, 244, 245, 246, 247], their application in CEC and related techniques is hampered by persistent shortcomings. First, the need for enantiomerically pure templates and lower resolution for structural analogs limits their generic use. Second, insufficient reproducibility, template leakage, tailing, and an uneven distribution of binding sites represent significant challenges in terms of column fabrication [243]. Lastly, the applicability of MIPs to aqueous real samples remains to be validated [246]. Efforts are being made toward new methods of preparation and standardized procedures, though improvement should come from a more thorough investigation of the general morphology of the formed stationary phase [245, 248, 249]. Further developments would also benefit from the commercial availability of several MIP capillaries [243]. Novel preparation methods including liquid‐crystalline networking and crowding could also prove useful in the near future [218, 245]. Furthermore, organic‐silica hybrid monoliths are of interest for MIP implementation as the integrity of the template cavities is kept mostly intact and the template removal is more straightforward [243]. To date, the best separation efficiency has been observed with OT‐CEC columns [250]. The further development of MIP columns is therefore expected to go alongside advances in the field of OT‐CEC.

#### 3.4.2 Metal‐organic frameworks

MOFs are a relatively novel class of microporous crystalline materials used in chiral analysis. These well‐defined three‐dimensional structures are highly ordered organic–inorganic hybrid composites consisting of metal ion nodes connected via multidentate organic linkers. Chirality is introduced either by using chiral auxiliary reagents during fabrication, through specific crystal growth from achiral components, or by adding chiral organic bridging ligands—the latter strategy being most commonly used [251, 252]. The potential of MOFs in chiral electrochromatographic analysis has been inspired by their adsorptive abilities in sample preparation and enantioselective extraction [251]. MOFs have demonstrated to be highly advantageous in terms of structural diversity, overall stability, and ease of fabrication. Since many different linker molecules can be incorporated in their structure, numerous MOF topologies can be created with the potential for both in‐pore functionality as well as outer surface modification [253]. Their straightforward design and crystallinity also allows for the effective tuning of their structural features including their pore size [253, 254, 255, 256]. Furthermore, their particular porous structure provides an accessible flow path [233] and shows a high surface area [254, 256, 257], while retaining excellent chemical, solvent, and thermal stability [253, 258]. Finally, fabrication costs depend on the type of fabrication procedure (i.e., purchasing commercially available MOFs or preparing the column in‐house) and the choice of chiral organic bridging ligand. Overall, simplicity of the MOF structure reduces costs, though at the expense of enantioselectivity. Nonetheless, higher fabrication costs are often compensated by the excellent reusability of the MOF columns, and the variety of flexible and relatively simple synthetic fabrication procedures make MOFs highly attractive for widespread application [258, 259].

Using MOFs as stationary phases in CEC has proven to be a viable approach for enantioselective analysis. Due to the promising results of preliminary studies on MOFs [251, 254], interest has grown exponentially along with the number of publications [260]; over 20 000 MOFs have now been reported and studied [261]. The original use of MOFs in solid‐phase microextraction [262] was rapidly followed by synthesis and controlled growth on bare silica supports [254, 257] and the use of novel chiral MOF columns in OT‐CEC [253, 263, 264]. Subsequently, further developments of chiral MOF columns were already directed toward improving fabrication procedures [256, 258, 265, 266].

**Figure 4:**
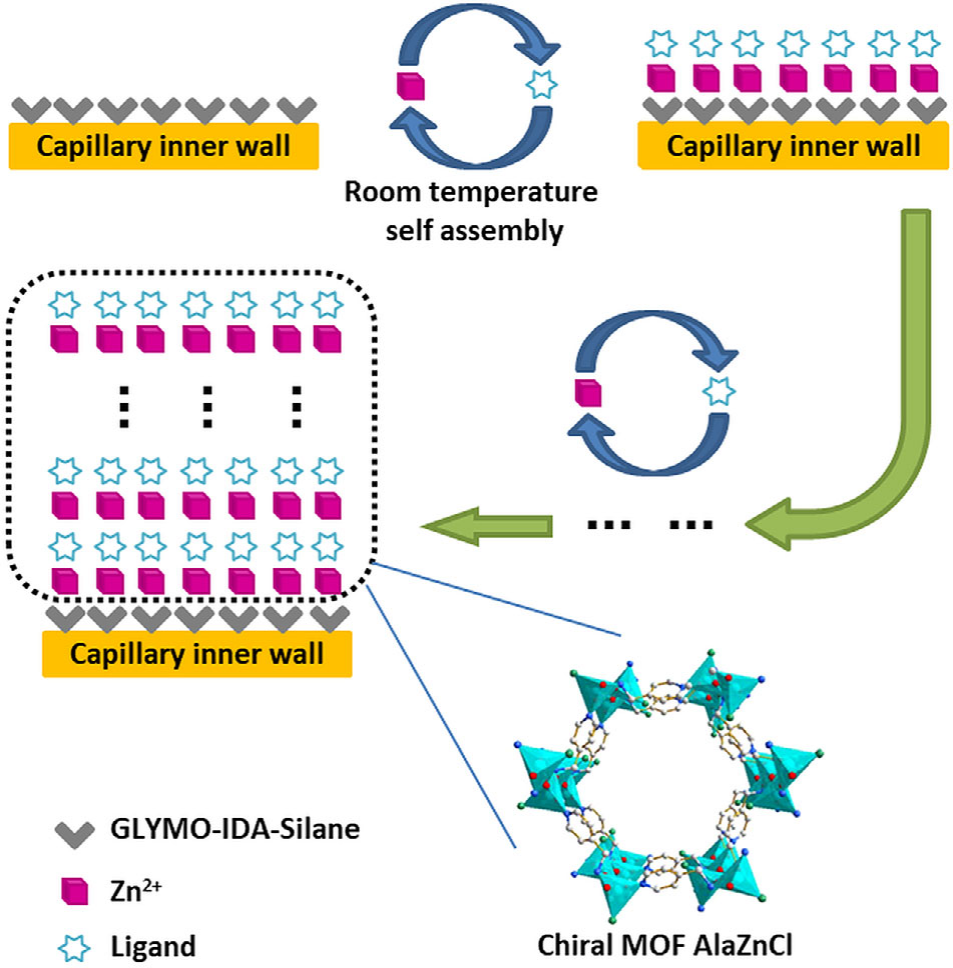
*In situ* layer‐by‐layer self‐assembly approach for the fabrication of a chiral metal‐organic framework (MOF) column. By cycling through treatments of Zn(CH_3_CO_2_)_2_·2H_2_O and 1‐HL solution, the chiral MOF column is generated at room temperature. The number of alternating cycles is used to determine layer thickness of the capillary wall. Adapted from [256] with permissions.

As an example, the*in situ* controllable synthesis developed by Pan et al. and illustrated in Fig. 4 presented the advantages of a layer‐by‐layer self‐assembly approach with mild preparation conditions at room temperature [256]. The MOF column fabrication process was then further simplified by the introduction of nucleating agents, reducing both the analysis time (<5 min) as well as the time needed to prepare the column (<5 h) [265]. Both MOF columns showed good enantioseparation of several monoamine neurotransmitters under optimized conditions and could be used for more than 100 runs while retaining their stability, reproducibility, and separation ability.

Ma and Ye et al. covalently attached MOF particles to a capillary wall as OT‐CEC stationary phase for the enantioseparation of amino acids [258, 266]. Their modification method could also be used with other MOF materials, broadening their scope in chiral analysis. Maya et al. offered a comprehensive overview of the different approaches developed for MOF immobilization on supports for analytical separation [267]. Lastly, the most recent studies on MOFs have focused on enhancing chiral resolution. A homochiral zeolite‐like MOF with a double‐helicity structure enabled an increase in the resolution of epinephrine and terbutaline enantiomers compared to the values reported in literature [259]. Furthermore, Sun et al. reported the synergistic application of an achiral MOF combined with carboxymethyl‐β‐CD as the chiral selector for the separation of five basic drugs [268]. The authors suggested a separation mechanism based on distinct interactions of slightly different CD‐analyte complexes with the pores and the extensive outer surface of the MOFs.

More fundamental research is required before extending the applications of MOFs. Systematic studies on the interactions between MOFs and various analytes should be prioritized to elucidate the chiral recognition mechanisms, where the large structural diversity and highly defined topology of MOFs represent significant advantages. Furthermore, their environmental impact in terms of safety and sustainability should also be investigated. Nonetheless, based on the rapid progress in the field of MOFs over the last years, exciting developments are expected to expand their use in OT‐CEC.

## 4 Electrodriven chiral separations coupled to MS

Chiral CE separations can greatly benefit from MS detection, particularly through a significant improvement in sensitivity. Moreover, high resolutions can be obtained with advanced mass analyzers [269]. However, the coupling between chiral CE and MS is very challenging due to the compatibility of current approaches in chiral EKC separations with conventional ionization methods. Although commercial CE‐MS interfaces have been developed, chiral EKC‐MS analysis requires additional practical adjustments of the separation approach to obtain MS‐compatible experimental conditions. Extensive research has provided several options to avoid ion source contamination with chiral selectors, including partial‐filling and counter‐migration techniques, and the use of molecular micelles. Since the chiral selectors are immobilized on the stationary phase in chiral CEC, this approach does not suffer from the same practical difficulties as chiral EKC‐MS. However, chiral CEC needs further method validation before further interfacing with MS.

### 4.1 Interfacing CE to MS

The hyphenation of CE with MS, most frequently using electrospray ionization (ESI), requires a dedicated interface to ensure both a suitable flow rate and the closure of the electrical circuit. Two CE‐MS interfaces are currently commercially available, namely, the sheath liquid and sheathless interfaces. In the sheath liquid interface, a sheath liquid (i.e., hydro‐organic mixture containing a small proportion of volatile acid or base) is added to the CE effluent, providing an adequate flow rate (μL/min range) for a stable electrospray. This interface is often preferred for large‐scale studies due to its higher reproducibility and stability, but may lead to lower sensitivities due to the dilution of the CE effluent [228]. In the sheathless interface, a modified capillary equipped with a porous tip is used in combination with a nanospray ionization source. Due to the absence of an additional sheath liquid, the sensitivities observed are typically higher, but this interface currently still suffers from a lower reproducibility and is more costly.

### 4.2 Chiral EKC‐MS

The contamination of the MS ionization source, leading to potential ion suppression, represents a considerable challenge in chiral EKC‐MS analysis, independently of the interface used. Indeed, without preventive measures, the PSPs will ultimately enter the MS source due to the EOF—if present—or by suction effect. Depending on their nature and concentration (stable crown‐ether complexes being one exception [270]), PSPs can significantly suppress the ionization of the analytes of interest, leading to severe loss of sensitivity and inaccurate (semi‐)quantitative results. Suppression of the EOF or lowering the concentration of the chiral selector can minimize these issues [271, 272], although without completely eliminating them. The partial‐filling technique (PFT) and the counter‐migration technique (CMT) are possible approaches to overcome these issues. With the PFT, the PSP is prevented from entering the MS system by decreasing its relative mobility or keeping it stationary within the capillary. With the CMT, the mobility of the PSP is reversed toward the capillary inlet. Both the PFT and the CMT have been successfully applied and they are now considered common practice within the field of chiral CE‐MS.

#### 4.2.1 Partial‐filling technique

**Figure 5:**
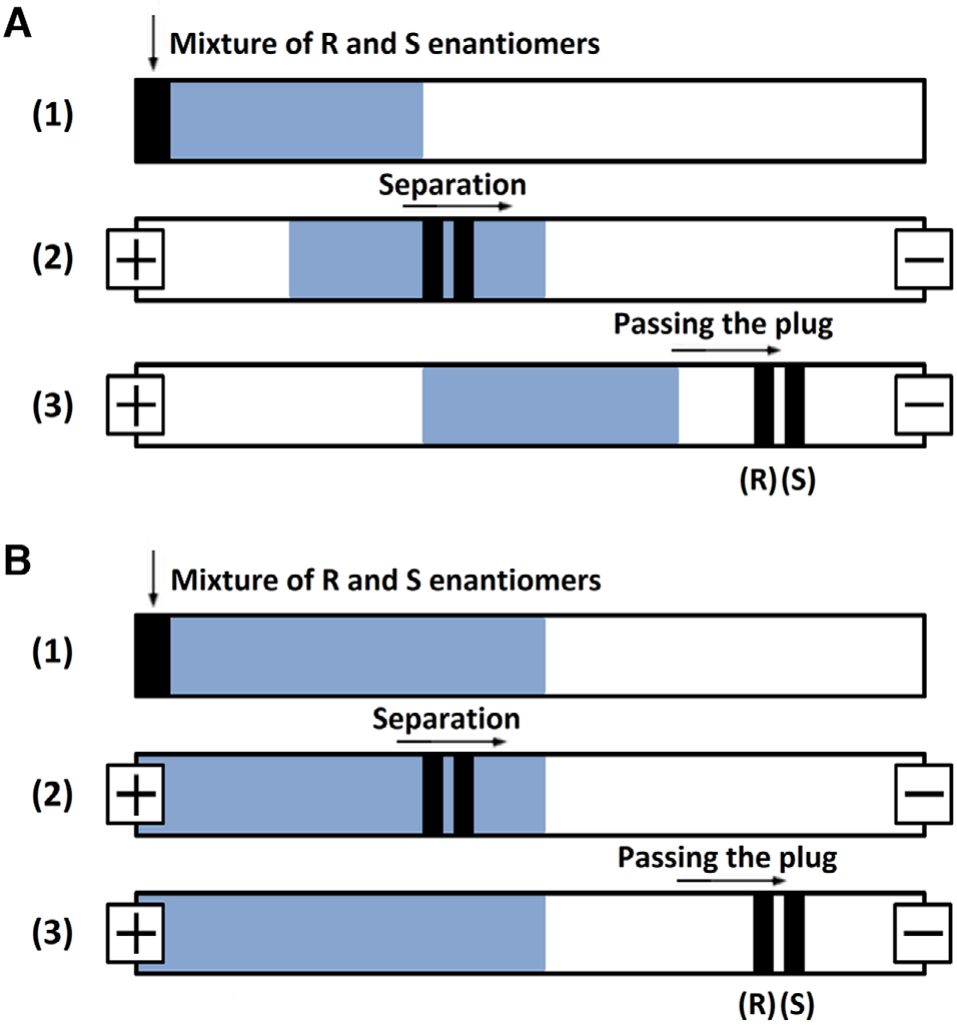
Partial‐filling technique applied to chiral separation of two enantiomers. (A) Illustration of the mechanisms observed with a selector plug with a lower mobility than the analyte. A racemic mixture of enantiomers enters the selector plug (1) where they are separated into two distinct bands (2). After passing through the selector plug, both enantiomers move with equal mobility allowing separate detection (3). (B) Illustration of the mechanisms observed with a stationary selector plug, with similar separation mechanics to schematic (A).

In the PFT, a small plug of the PSP is introduced into the capillary instead of being incorporated in the bulk buffer solution. The analytes first interact with the PSP in the selector plug prior to their migration toward the detector, while the PSP stays within the capillary and does not enter the ion source. An apparent mobility higher than the mobility of the EOF is required for the analytes to pass the selector plug (Fig. 5A). If this cannot be achieved, a stationary selector plug can be employed (Fig. 5B), where the EOF is suppressed by using coated capillaries or by using a BGE at low pH. Longer migration times are observed for the latter strategy; however, for both approaches, an optimized concentration and zone length result in an increased separation efficiency and sensitivity. Usually, slightly lower resolutions are obtained with the PFT compared with the CMT due the shorter zone length and possible zone broadening at the PSP‐buffer boundary.

The PFT was first implemented in EKC‐UV set‐ups to counter the background absorbance for UV detection, but is nowadays also well adapted for EKC‐MS. Valtcheva et al. [273] described the first use of the PFT for free solution CE in 1994, followed by modifications from Tanaka et al. [274] in 1995 that enhanced peak resolution and reproducibility. The first literature to report the PFT in combination with EKC‐MS was from Javerfalk et al. [275] in 1999, followed by Tanaka et al. [276] who employed the PFT for EKC‐MS with crown ether chiral selectors in 2000. The PFT can now be easily integrated into commercial CE‐MS interfaces and automated for routine analysis [277]. Due to the relatively small plug length, the consumption of chiral selector is reduced, enabling the use of more exotic or expensive variants and broadening the scope of PSP‐analyte interactions. Furthermore, nonvolatile PSP can also be introduced into the system.

Promising results using the PFT in EKC‐MS have been reported. For instance, Sánchez‐López et al. developed a chiral EKC methodology with ion trap MS (EKC‐IT‐MS) for quality control of the commercialized single‐isomer duloxetine, where the nonvolatile (2‐hydroxypropyl)‐β‐CD was prevented from entering the mass spectrometer by introducing a selector plug of 38% of the total capillary length [79]. In another study by Lee et al., native amino acids were separated employing (+)‐(18‐crown‐6)‐2,3,11,12‐tetracarboxylic acid in an aqueous buffer using a selector plug that filled 70% of the capillary [278]. Enantiomers were identified by quadrupole TOF/MS and no peaks corresponding to the chiral selector were observed. Alternatively, Xia et al. found that a filling volume of 30.6% was optimal for the enantioseparation of chiral dipeptides by EKC‐MS employing a chiral crown ether [279]. Strongly acidic conditions and a low EOF were maintained to keep the crown ether neutral and prevent its migration toward the capillary outlet. Finally, Sanchez‐Hernandez et al. demonstrated the simultaneous separation of 17 FMOC‐derivatized amino acids in EKC using the antibiotic vancomycin as the PSP [124]. The selector plug was introduced at 50 mbar for 150 s; filling times were optimized against the effective capillary length. Amino acids present in overlapping peaks could be successfully discriminated using IT‐MS.

#### 4.2.2 Counter‐migration technique

In the CMT, the electrophoretic mobility of the PSP is reversed, causing it to migrate away from the detector. Indeed, if the electrophoretic mobility of the PSP is sufficiently higher and in the opposite direction of the EOF, its effective mobility will be directed toward the capillary inlet. Highly charged PSPs are therefore required, typically sulfated β‐CDs [280, 281, 282]. The earliest description of the CMT comes from Schulte et al. [283], who in 1997 used CD derivatives to separate neutral chiral analytes. This was followed by important work from Iio et al. [284], who also used the CMT with modified CDs for chiral separation of methamphetamine and its metabolites from urine. More recently, Piestansky et al. successfully used an ionizable countercurrent migrating CD combined with a complex multidimensional isotachophoresis‐CE‐MS system (Triple Quadrupole), illustrating the potential of charged PSPs [285]. Alternatively, Svidrnoch et al. demonstrated the use of cathodically migrating vancomycin for the separation of a chiral anionic biomarker important for the diagnosis of neurometabolic disorders. In their approach, the solution of chiral selector in the BGE filled the whole capillary. Contamination of the ion source at the capillary outlet was prevented by keeping the MS ion source in “waste” mode during filling and sample injection. Furthermore, Moini et al. demonstrated the combined use of the PFT and the CMT for the analysis of synthetic cathinones and their optical isomers; although both techniques could be applied in conjunction, better results were obtained with the CMT only [280]. An important aspect during method development is the selection of a suitable charged PSP that can efficiently interact with the analyte. The enhanced separation window and possible ionic bonds between the analyte and the charged PSP can contribute to chiral resolution. However, interactions should not be too strong as the analyte will be carried back by the PSP and will not be detected [80]. Furthermore, opposite charges of the PSP and chiral analyte are required to avoid mutual repulsion.

#### 4.2.3 Chiral MEKC‐MS

Currently, the use of conventional micelles in MEKC‐MS is limited. Similarly to chiral selectors, a decrease in ionization efficiency and possible contamination of the ion source are important drawbacks when using conventional micelles. Indeed, the dissociation of the micelles in the ion source results in a high abundance of nonvolatile low molecular weight surfactant ions that suppress the ionization of the analytes and contaminate the MS inlet [286]. Moreover, their high surface activity lowers the stability of the electrospray [287]. For these reasons, traditional chiral surfactants that would migrate to the MS inlet are not feasible.

In order to overcome these challenges, several approaches for on‐line coupling have been developed that either focus preventing the PSP from entering the mass spectrometer, or using other types of micelles. The latter represents the area where most of the development has taken place over the last years, particularly in using volatile surfactants and polymeric micelles. Three recent studies described the use of the volatile achiral surfactant ammonium perfluorooctanoate as micellar PSP for the enantioselective analysis of several amino acids with MS detection [180, 288, 289]. For this purpose, amino acids were derivatized by the chiral reagent (±)‐1‐(9‐fluorenyl)ethyl chloroformate. As an example, Moldovan et al. developed a fully automated MEKC‐MS method for the analysis of chiral amino acids in artificial cerebrospinal fluid samples with good reproducibility and linearity [180]. Furthermore, Moreno‐González et al. also demonstrated the hyphenation of MEKC with MS/MS by using a sheathless interface with ammonium perfluorooctanoate as additive. However, this approach was applied only for the analysis of achiral analytes [290].

Polymeric micelles, often referred to as molecular micelles, are polymeric structures that share similar chiral recognition to surfactant‐based micelles, but are compatible with MS detection as they do not degrade into monomers when introduced into the ion source. These fixed micellar structures have covalent bonds that are difficult to ionize during the ESI process, which hampers their entrance in the mass analyzer [291]. Moreover, molecular micelles can be used at any concentration in their solubility range, which results in a lower surface concentration in the electrospray droplet and thus a more stable electrospray [286]. They are stable in the presence of organic solvents [286, 292] and over a wide pH range, and their structure is highly customizable. For instance, different parts of the micelle can have hydrophobic, hydrophilic, charged, or chiral properties, and each part can be optimized for the interaction with a specific chiral analyte. Moreover, their assembly can take on many different shapes and sizes, where more rigid micelles can bring unique selectivity toward analytes of interest.

Much of the research to date has focused on polymers derived from undecylenic acid, with carbamate and amide linkages functionalized with amino acids [293]. Early studies demonstrated the different selectivity profiles of various polysodium *N*‐undecanoyl and *N*‐undecenoxycarbonyl PSPs, as well as the superiority of MEKC‐MS to MEKC‐UV in terms of sensitivity [294, 295]. Rizvi et al. developed a quantitative MEKC‐MS assay employing sulfated amino acid derived molecular micelles for the enantioseparation of (±)‐pseudoephedrine [287]. Operating at low pH (i.e., pH 2.0), a LOD of 325 ng/mL could be achieved. Five additional studies by Shamsi and co‐workers reported the potential of similar polymeric micellar PSPs for the separation of different chiral analytes [149, 296, 297, 298, 299, 300]. More recently, new information regarding the chiral recognition of amino acid based molecular micelles has become available, illustrating the growing interest for MEKC [301]. Molecular dynamics suggested the key importance of intermolecular stereoselective hydrogen bonding. Given the unexplored potential of all possible micellar assemblies, research within the field of chiral MEKC‐MS is expected to continue toward novel polymeric PSPs and possible applications.

### 4.3 Chiral CEC–MS

The benefit of chiral CEC‐MS as compared to chiral EKC‐MS is linked to the immobilization of the chiral selectors on the column, therefore minimizing ionization suppression and chemical noise caused by the presence of selectors in the BGE. The first researchers to couple CEC to MS were Verheij et al. in 1991 [302], who demonstrated its feasibility when combined with fast atom bombardment for ionization. Today, however, ESI is the more common ionization mode for CEC‐MS. Generally, the flow rate is too low for a stable electrospray and a sheath‐liquid interface is required for MS coupling [209, 293]. With the aid of a low‐flow electrospray or a nanospray device, the sheathless interface can also be used [228]. Nevertheless, further research in chiral CEC‐MS would benefit from a dedicated CEC‐MS interface, which remains to be commercialized [303].

Following the first use of a chiral packed column for CEC‐MS from von Brocke et al. in 2002 [304], a small number of reports of successful enantioseparation by packed‐column CEC‐MS have been published. As an example, Zheng et al. separated warfarin and the internal standard coumachlor on a 5 μm (3*R*,4*S*)‐Whelk‐O1 stationary phase packed on silica support [305]. Furthermore, two vancomycin functionalized stationary phases were employed for the simultaneous separation of β‐blockers by CEC‐MS [306, 307]. Both stationary phases were retained within the capillary by in‐house tapering and burning an additional inlet frit. The study reported excellent durability of sulfated and sulfonated polysaccharide packed capillaries, as well as reproducible enantioselectivity using different mobile phases after CEC‐MS analysis of aminoglutethimide [308].

Monolithic and OT‐CEC can both be readily coupled to a mass spectrometer. Since the stationary phase is fixed within the capillary, no installing of frits or tapering is required. In the field of chiral monolithic CEC‐MS, a few studies have been reported. As an example, the methacrylate‐β‐CD monolith developed by Gu et al. demonstrated excellent stability, reproducibility of retention time, and enantioselectivity of in a CEC‐MS approach, using hexobarbital as a model chiral analyte [227]. Schurig and Mayer [202] also showed the potential of chiral OT‐CEC‐MS, coating the open capillary with CDs to perform chiral separation of drugs in 2001. Additionally, Kitagawa et al. prepared avidin‐functionalized fused silica capillaries for the enantioseparation of abscisic acid and several arylpropionic acids by OT‐affinity‐CEC with sheath liquid MS detection [232].

## 5 Conclusions and future perspectives

Chiral separation has been a highly dynamic field of research in recent years with many novel techniques, additives, and stationary phases developed to improve the enantioseparation of a variety of chiral molecules. In EKC, recent efforts have led to significant advances in the use of single and dual selector systems employing chiral selectors, molecular micelles, and derivatization agents. CDs, crown ethers, and antibiotics are commercially available and can be readily employed, while chiral ILs represent a promising approach to further tailor the selectivity when added to the BGE. In this context, a better understanding of the chiral recognition between analytes and PSPs will be highly beneficial to facilitate the selection of suitable PSP–analyte combinations and reduce trial‐and‐error approaches.

Significant progress has also been observed in the field of packed‐column, monolithic, and OT‐CEC, despite remaining practical challenges and lack of properly validated methods. Future research should focus on expanding the chiral monomer library for monoliths, improving the fabrication procedures of existing stationary phases, and developing standardized methods. Further efforts in the field of CEC are expected toward the development of novel stationary phases and establishing the use of MIPs and MOFs.

Chiral CE‐MS has demonstrated its potential for the enantioseparation of a wide variety of compounds present in samples of variable complexity. Chiral analytes with a large diversity of physicochemical properties are efficiently separated using these approaches, including both neutral and charged molecules. The PFT and the CMT have been a significant step to enable the coupling of EKC to MS, fostering its use in many different fields. Biomedical, clinical, and pharmaceutical research on biologicallyactive enantiomers can highly benefit from adopting chiral CE‐MS into routine analysis; chiral CE‐MS is also expected to play an important role in forensic analytical toxicology and metabolomics, as well as in food and agrochemical industry. The implementation of chiral CE‐MS will strongly benefit from further technological developments in CE‐MS instrumentation, as well as novel approaches in the developments of reproducible and MS‐compatible conditions.

Chiral CE and mainly chiral CE‐MS remain far from being fully accepted as state‐of‐the‐art analytical techniques in many academic and industrial environments. Nonetheless, the current and future developments discussed in this review are expected to foster the use of electrodriven separation techniques in the context of chiral analysis.

*The authors have declared no conflict of interest*.
